# Artificial intelligence in outpatient management: a simulation-based study in a Chinese tertiary hospital

**DOI:** 10.3389/frai.2025.1696586

**Published:** 2026-01-05

**Authors:** Xiaoxiao Quan, Kejun Wang, Junjie Pan, Zhiyu Fang

**Affiliations:** 1 Shenzhen Second People's Hospital, First Affiliated Hospital of Shenzhen University, Shenzhen, China; 2 School of Political Science and Public Administration, Wuhan University, Wuhan, China; 3 Shenzhen Polytechnic University, Shenzhen, China

**Keywords:** artificial intelligence, Chinese tertiary hospital, deep learning, resource optimization, simulation study

## Abstract

The Chinese healthcare system faces significant challenges such as an aging population and uneven resource distribution, necessitating technological innovations to enhance service efficiency and quality. This study explores the application, potential value, and challenges of artificial intelligence (AI) in outpatient management in China using simulated data and a physician survey. Simulation results, based on a comparison between an LSTM model and a traditional ARIMA model, demonstrate that the deep learning-based approach outperforms in predicting outpatient flow, improving forecasting accuracy by approximately 15%. Under simulated conditions, AI implementation reduced patient waiting times by about 30% and increased doctors’ work efficiency and satisfaction, as supported by survey responses. These findings suggest that AI can optimize resource allocation and patient experience in outpatient settings. However, this study is primarily based on simulation, and real-world applicability may be limited. Additional concerns regarding data privacy, regulatory compliance, and physician acceptance remain critical.

## 1 Introduction

Over the past decade, healthcare systems worldwide have faced numerous challenges. With the accelerating trend of population aging and rapid advancements in medical technology, outpatient volumes at hospitals have continued to rise, leading to longer patient waiting times, increased work pressure on medical staff, and strained medical resources (Brendel et al., 2021; Sahu et al., 2021). Traditional outpatient management models are no longer sufficient to meet the needs of modern healthcare institutions, necessitating the adoption of information technology and intelligent solutions to enhance the efficiency of medical services. The emergence of artificial intelligence (AI) has brought unprecedented opportunities to the medical field (Chong et al., 2020; Li et al., 2021; Li et al., 2022; Harada and Shimizu, 2020; Chew and Achananuparp, 2022; Manickam et al., 2022; Masoumian Hosseini et al., 2023; AlMuhaideb et al., 2019; Incze et al., 2021). AI technology has achieved significant results in medical image analysis, gene sequencing, and disease prediction, providing new approaches to medical diagnosis and treatment. In particular, the outstanding performance of deep learning technology in big data processing and pattern recognition is considered to be the key to solving complex problems in the medical field.

In the field of outpatient management, AI is increasingly widely used. For example, through deep learning algorithms, healthcare organizations can more accurately identify abnormalities in medical images, improve diagnostic accuracy and speed up the diagnosis and treatment process. In addition, AI has shown great potential in the direction of gene sequencing and personalized treatment (Karim et al., 2021). Moreover, by analyzing patients’ past medical history, lifestyle and genetic information, AI models can achieve disease risk prediction and provide a basis for early intervention (Kijowski et al., 2020). With the rapid development of technology, AI has become an integral part of the modern healthcare system, especially showing an important role in hospital outpatient management.

Traditionally, outpatient management mainly relies on manual methods to process data and schedule resources, with limited efficiency. In recent years, many hospitals have begun to introduce AI technology to improve service quality and address resource constraints. However, systematic studies evaluating the effectiveness of AI in outpatient management—particularly in the Chinese context—remain scarce. Existing research often lacks empirical validation, and real-world data are difficult to obtain due to privacy and regulatory constraints. This gap is especially evident in China, where regional disparities and a rapidly aging population create unique operational challenges. To address this, the present study employs simulated data—a necessary approach given limited access to real clinical datasets—and combines it with survey analysis to objectively assess the potential of AI in patient flow prediction and resource optimization within Chinese outpatient settings.

## 2 Application of artificial intelligence in outpatient management

### 2.1 Data analysis and prediction

AI plays an important role in outpatient management through advanced data analytics and predictive techniques. Using deep learning and machine learning algorithms, AI systems are capable of analyzing large volumes of outpatient-related information, including consultation timestamps, department identifiers, historical visit patterns, waiting times, and other operational indicators. Compared with traditional statistical approaches, AI methods better capture nonlinear relationships and temporal dependencies, which are essential for predicting fluctuations in patient flow.

This study was conducted in a Class III Grade A tertiary teaching hospital located in Shenzhen, China, which provides comprehensive outpatient services with an annual volume of approximately 3.2 million visits. As a major regional referral center serving southern China, the hospital covers a wide range of specialties, including internal medicine, surgery, pediatrics, and emergency care. Two types of data were used in the analysis. First, anonymized historical outpatient registration records from January 2023 to December 2024 were used to train and validate the LSTM and ARIMA forecasting models. These records included consultation timestamps, patient counts, and departmental information. Second, because real operational testing could not be conducted within the hospital setting, a simulated dataset was constructed to evaluate model performance and system behavior under controlled and scalable conditions. The simulation parameters—including patient arrival patterns, department-level demand fluctuations, and staffing constraints—were derived from summary statistics of the real dataset. The simulation model followed a discrete-event structure in which patient arrivals were represented by a non-homogeneous Poisson process fitted to empirical arrival curves. Service time distributions were estimated using departmental averages and variance measures. Model validity was confirmed by ensuring that simulated patient flow patterns matched the real dataset within a ± 5% deviation during peak and off-peak periods.

Based on these datasets, we developed the LSTM model as the primary prediction framework and used the traditional ARIMA model as a benchmark. The real dataset was randomly divided into a 70% training set and a 30% testing set, and predictive accuracy was assessed across morning, afternoon, and evening periods. To complement the modeling work, a survey was conducted among 30 physicians from internal medicine, surgery, and pediatrics to evaluate perceived usefulness and workflow impact of AI-assisted scheduling. The questionnaire consisted of 10 items rated on a 5-point Likert scale. Its clarity and content validity were reviewed by two senior clinicians and pilot-tested with five physicians before final deployment.

The prediction results, summarized in Table 1, show that the LSTM model consistently outperformed ARIMA across all time periods, with an average accuracy improvement of approximately 15%. During the morning peak, the LSTM model achieved around 90% accuracy compared to roughly 80% with ARIMA. These findings demonstrate that deep learning approaches can better capture complex variations in outpatient flow and provide hospitals with more reliable forecasts for resource planning and allocation.

**Table 1:** Prediction accuracy comparison: AI vs. traditional methods.

Time period	Traditional accuracy (%)	AI accuracy (%)
Morning	80	90
Afternoon	75	88
Evening	70	85

### 2.2 Real-time resource optimization

Outpatient flow is influenced by seasonal illnesses, sudden public health events, and fluctuations in healthcare resource availability, making real-time resource optimization essential for improving operational efficiency and enhancing the patient experience. In this study, we evaluated the potential of an AI-based scheduling and resource allocation approach under simulated conditions to explore how real-time adjustments may support outpatient operations in a dynamic environment. The AI system continuously monitored simulated patient arrivals and provided recommendations based on available staffing and departmental capacity. When a rapid increase in simulated patient volume occurred, the system generated alerts that allowed administrators to hypothetically reallocate staff or extend outpatient service hours, demonstrating how AI-assisted decision support could help mitigate congestion in real settings.

The simulation incorporated intelligent scheduling, in which the AI system generated optimized appointment and staffing plans using information on physicians’ working hours and specialty distributions. These optimized schedules aimed to improve workflow continuity and patient throughput. To assess potential effects on operational performance, we compared average patient waiting times before and after the introduction of the AI-assisted scheduling strategy within the simulated environment. As shown in Table 2, simulated waiting times decreased across all departments after AI scheduling was applied—for example, from 45 to 30 min in internal medicine, from 50 to 35 min in surgery, and from 40 to 25 min in pediatrics. Hospital-wide average waiting time was reduced from 42 to 28 min. These results represent percentage reductions of approximately 30–37%, although no statistical significance testing was performed and confidence intervals were not calculated. Therefore, these findings should be interpreted as preliminary and descriptive rather than inferential.

**Table 2:** Change in patient waiting times after AI implementation.

Department	Pre-AI waiting time (min)	Post-AI waiting time (min)	Reduction (%)
Internal Medicine	45	30	33%
Surgery	50	35	30%
Pediatrics	40	25	37.5%

To complement the simulation analysis, we conducted an exploratory survey of 30 physicians from internal medicine, surgery, and pediatrics to assess perceived usefulness and workflow impact of the AI-assisted scheduling system. Responses were collected using a 5-point Likert scale. The average satisfaction score was approximately 4.1, and physicians reported an estimated 20% increase in daily consultations. Given the small sample size and self-reported nature of the responses, the survey findings should be viewed as indicative rather than generalizable. Nonetheless, the results provide initial insights into clinicians’ acceptance and perceived benefits, supporting the feasibility of future large-scale evaluations (Table 3).

**Table 3:** Physician feedback on AI scheduling system.

Title	Satisfaction (Avg, /5)	Efficiency increase (%)
Chief Physician	4.3	+25%
Attending	4.1	+20%
Resident	3.8	+15%

## 3 The potential and challenges of AI in outpatient management

While AI shows great potential in outpatient management, its widespread use faces a number of challenges. First, data privacy and security issues are particularly critical. Hospitals introducing AI technology must ensure the security of patients’ personal medical data and take strict measures to prevent unauthorized access or misuse. At the same time, AI system applications also need to comply with relevant regulatory requirements to ensure legal compliance in the use of medical data. For example, the physician survey in this study found that about 20% of the responding physicians were skeptical about the results of the system’s algorithms at the initial stage of use, and were concerned about data leakage and privacy protection issues. This suggests that in practical application, hospitals must establish a sound data security mechanism and formulate appropriate laws and regulations to protect patients’ rights and interests.

Second, although AI can provide valuable predictions and suggestions, the professional judgment and experience of doctors are still indispensable. Therefore, how to effectively combine AI technology with doctors’ expertise has become a major challenge. Doctors need to learn to correctly understand and apply the data and recommendations provided by AI, and integrate them into final treatment decisions. At the same time, hospital administrators need to adapt to this technological change and learn to use AI systems appropriately in outpatient management. This is also reflected in the results of the questionnaire survey mentioned above: although most physicians rate the AI effect positively, a small number of physicians still need time to familiarize themselves with and trust the system’s decision-making. These issues need to be addressed through continued exploration and training.

In conclusion, the application of AI in outpatient management is promising, but it is also accompanied by multiple challenges such as data security, regulatory compliance, and doctor-patient cooperation. Only on the premise of continuously improving technology, processes and management can the potential of AI be fully utilized to truly enhance the quality of healthcare services.

## 4 Discussion

The application of artificial intelligence (AI) in outpatient management is gradually demonstrating its potential to enhance healthcare service efficiency. The findings from this study, conducted through simulation-based modeling and survey analysis, indicate that AI technologies offer measurable improvements in both patient flow prediction and real-time resource optimization. By leveraging deep learning techniques, AI models were able to capture complex nonlinear trends in outpatient data, achieving a forecasting accuracy approximately 15% higher than that of traditional statistical methods. Such predictive advantages may support hospitals in planning staffing needs more proactively and improving the continuity of care.

Similarly, the results of the simulated scheduling experiments suggest that AI-enabled real-time resource optimization can reduce patient waiting times and enhance physicians’ perceived work efficiency. These findings highlight the potential value of AI-driven dynamic scheduling in reducing bottlenecks and enhancing the overall patient experience in outpatient settings. However, these improvements must be interpreted with caution. Since the present study relied predominantly on simulated data and a small-scale physician survey, the observed effects may not fully reflect real-world operational conditions. Outpatient environments differ significantly across regions, institutions, and health systems, and variations in patient demographics, data quality, staffing structures, and workflow practices may influence AI performance. Future validation using real-time hospital data and larger, multi-center investigations will therefore be essential to confirm the generalizability of these findings.

In addition, the discussion of AI implementation must consider several practical challenges. First, the use of simulated datasets—necessitated by limited access to real clinical data due to privacy regulations—presents inherent limitations, particularly in representing the complexity and variability of real patient behaviors. Second, international experiences suggest that successful integration of AI into outpatient workflows requires substantial investment in digital infrastructure, interdisciplinary coordination, and ongoing staff training. Countries such as the United States, Singapore, and South Korea have reported that barriers to AI adoption often include cost constraints, data interoperability issues, and resistance among clinical staff due to unfamiliarity with algorithmic decision-making. These factors are equally relevant in the Chinese healthcare context.

Lastly, while most physicians in our survey expressed positive attitudes toward AI-enabled scheduling, the sample size was small and should be considered exploratory. Concerns regarding system reliability, transparency, and long-term sustainability remain. Thus, addressing data privacy, regulatory compliance, and algorithmic explainability will be critical for achieving broader acceptance among healthcare providers.

In summary, although the simulation-based findings support the potential benefits of AI in outpatient management, further research in real clinical environments is needed to validate these results. Future work should incorporate multicenter datasets, evaluate cost-effectiveness, and explore training programs to support frontline staff in adopting AI systems. Continuous improvement of AI algorithms and stable infrastructure development will be crucial for ensuring the safe, equitable, and sustainable integration of AI into outpatient care.

## 5 Limitations of the study

This study has several limitations that should be acknowledged. First, the analysis relied primarily on simulated data constructed from summary statistics of a single tertiary hospital, which may not fully capture the complexity and variability of real-world outpatient operations. Second, the study was conducted in only one hospital setting, limiting the generalizability of the findings to other institutions with different service structures, patient demographics, or resource constraints. Third, the physician survey included a relatively small sample of 30 respondents, which restricts the strength of inferences about staff acceptance and may be subject to response bias. Fourth, the survey data were self-reported, which may introduce subjective bias and overestimation of perceived improvements in workflow efficiency. Finally, the study lacks external validation through multicenter trials or real-world implementation, and therefore the findings should be interpreted as exploratory. Future research involving larger datasets, multiple hospital settings, and prospective real-world evaluations will be essential to confirm the robustness and applicability of the proposed AI-assisted outpatient management strategies.

## 6 Conclusion and outlook

Artificial intelligence (AI) technology provides promising and innovative tools for outpatient management and has the potential to substantially improve operational efficiency and patient experience. In this study, simulation-based experiments and a physician survey were used to explore the feasibility of AI-assisted prediction and scheduling systems. The findings suggest that deep learning–based models can enhance the accuracy of outpatient flow forecasting, and AI-driven scheduling may help reduce patient waiting times while improving physicians’ work efficiency.

However, these results should be interpreted as exploratory. Because the study relied on simulated datasets and a limited physician sample, the findings cannot be directly generalized to real-world clinical settings. Outpatient management in practice involves more complex conditions, including regulatory requirements, ethical concerns, data privacy considerations, heterogeneity in hospital workflows, and variability in patient behavior. These factors may influence the actual performance and acceptance of AI systems.

Future studies should therefore include larger, multicenter trials and real-world validation to further test the robustness and generalizability of AI-assisted outpatient management tools. Continued optimization of AI algorithms, improved system–clinician integration, and strengthened governance frameworks will be essential to ensure that AI technologies can be safely and effectively deployed at scale, ultimately contributing to more efficient healthcare delivery and improved patient outcomes.

## Data Availability

The original contributions presented in the study are included in the article/supplementary material, further inquiries can be directed to the corresponding author/s.
